# Mechanisms of thermoregulation in *Pseudomonas aeruginosa*

**DOI:** 10.1128/jb.00495-25

**Published:** 2026-03-11

**Authors:** Rachel E. Robinson, Joanna B. Goldberg

**Affiliations:** 1 Microbiology and Molecular Genetics Program, Graduate Division of Biological and Biomedical Sciences, Laney Graduate School, Emory University 310203, Atlanta, Georgia, USA; 2 Division of Pulmonary, Asthma, Cystic Fibrosis, and Sleep, Department of Pediatrics, Emory University School of Medicine 209740, Atlanta, Georgia, USA; 3 Emory+Children’s Center for Cystic Fibrosis and Airway Disease Research, Emory University School of Medicine 12239, Atlanta, Georgia, USA; University of California San Francisco, San Francisco, California, USA

**Keywords:** thermoregulation, *Pseudomonas aeruginosa*, heat shock, RNA thermometers

## Abstract

*Pseudomonas aeruginosa* is a highly versatile opportunistic pathogen and a major cause of acute, chronic, and even lifelong respiratory infections in people with cystic fibrosis. It can also cause corneal infections, burn/wound infections, and bacteremia. *P. aeruginosa* is often found in human-associated environments such as hospitals, where it is a frequent cause of nosocomial catheter-associated urinary tract infections and ventilator-associated pneumonia. As a nosocomial pathogen, a major environmental change associated with transmission is the change from room or ambient temperature to human body temperature. *P. aeruginosa* is highly studied for its regulatory and adaptive responses to environmental stimuli, such as low iron conditions or the presence of antibiotics, but temperature regulation, or thermoregulation, is relatively understudied, particularly at a mechanistic level. This review explores the current understanding of mechanisms of global, transcriptional, post-transcriptional, and post-translational thermoregulation in *P. aeruginosa*, with a discussion on gaps in the field’s knowledge and directions for future research. More mechanistic studies of thermoregulation inspired by the open questions presented here will improve our understanding of how *P. aeruginosa* adapts to different temperatures.

## *PSEUDOMONAS AERUGINOSA* IS AN IMPORTANT OPPORTUNISTIC AND NOSOCOMIAL PATHOGEN

*Pseudomonas aeruginosa* is a highly adaptable gram-negative opportunistic and nosocomial pathogen (1). Although it can survive in natural environments like soil or water, it is most often isolated from human-associated environments, such as hospital sinks and surfaces or polluted natural sites (2, 3). *P. aeruginosa* is intrinsically resistant to many antibiotics due to low membrane permeability, efflux pumps, and growth in biofilm structures (4–6) and can readily evolve or acquire genomic elements that confer additional antibiotic resistance (7, 8). Multidrug-resistant (MDR) *P. aeruginosa*, particularly with carbapenem resistance, is classified as a serious public health threat by the Centers for Disease Control and Prevention (CDC) (9), and the incidence of MDR *P. aeruginosa* in healthcare settings has increased since 2019 (10).

*P. aeruginosa* has long been studied as a cause of acute and chronic respiratory infections in people with the genetic disorder cystic fibrosis (CF), which is caused by mutations in the cystic fibrosis transmembrane conductance regulator, a chloride and bicarbonate channel that is required to maintain levels of salt and water throughout the body (11). CF is a multi-organ disease with many complications, one of which is the build-up of thick mucus in the lungs (12), which is an ideal environment for bacterial growth. Recent research has revealed that CF respiratory infections are often polymicrobial, and these infections are associated with worse clinical outcomes (13, 14). However, *P. aeruginosa* remains a major cause of morbidity and mortality for people with CF, as it is particularly adept at adapting to the CF respiratory environment and causing chronic infections that may last decades, leading to significant respiratory decline (15–18). In addition to respiratory CF infections, *P. aeruginosa* can cause nosocomial infections (19), such as ventilator-associated pneumonia (20), catheter-associated urinary tract infections (21), infections in burns and wounds (22), and bacteremia (23), as well as ocular infections in scratched or compromised corneas (24).

**Fig 1:**
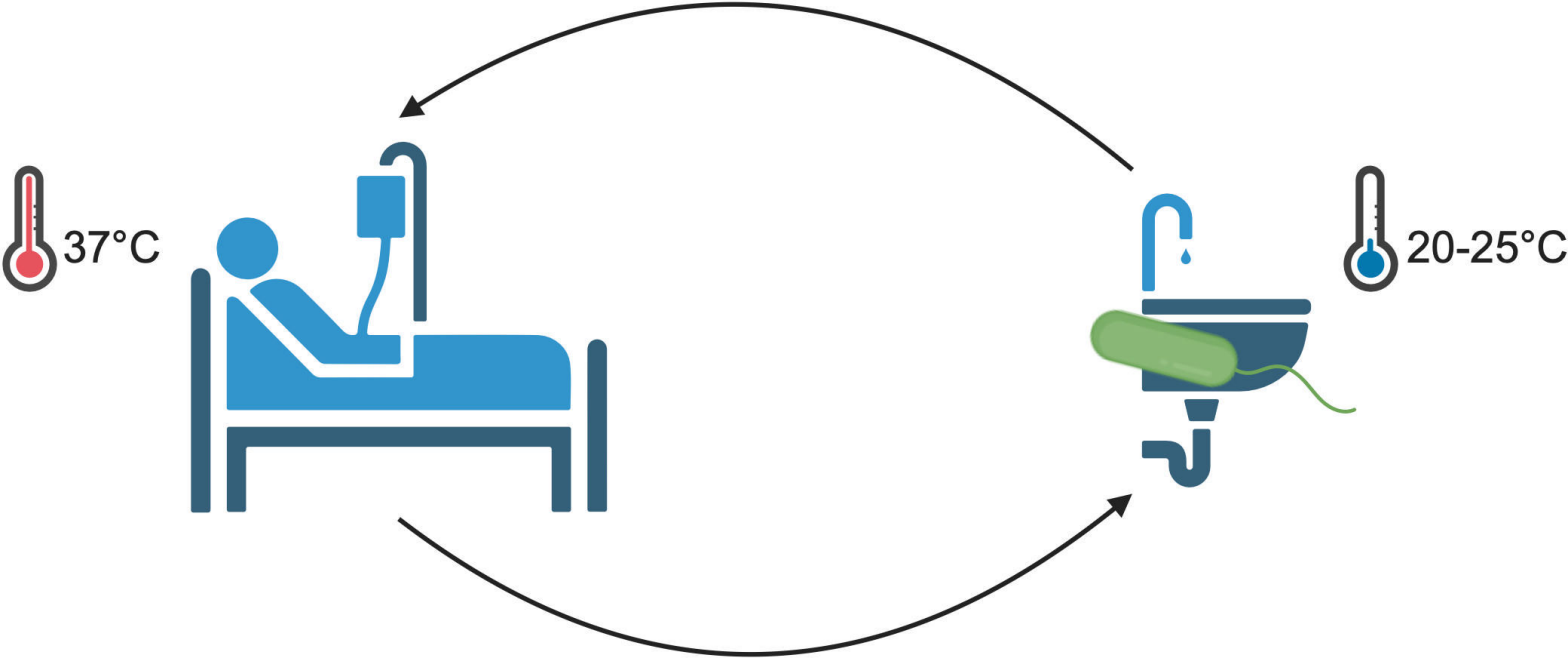
*P. aeruginosa* traverses environments of different temperatures as an opportunistic and nosocomial pathogen. *P. aeruginosa* can reside on and in hospital surfaces, depicted here by a contaminated sink. In this environment, *P. aeruginosa* experiences ambient (room) temperature, often 20°C–25°C (right). However, upon encountering the human host, *P. aeruginosa* must adapt to an environment at 37°C (left). Transmission back to the environment from an infected individual again involves a temperature shift to room temperature. Created with BioRender.com.

Although *P. aeruginosa* can survive and grow across a broad range of temperatures from 4°C to 42°C (25), it is commonly studied in the laboratory setting at 37°C (26). This temperature best models that of the human body and facilitates the optimum growth rate for *P. aeruginosa* (26). Consequently, most of the laboratory studies conducted on the regulation of phenotypes important for pathogenesis—quorum sensing, biofilm formation, and virulence factor production—have been conducted at 37°C and much less is known about *P. aeruginosa* physiology at lower or higher temperatures. Moreover, as *P. aeruginosa* is an opportunistic and nosocomial pathogen, it exists in the environment and survives at the temperature of that environment before encountering the human host. This transition between room or ambient temperature (20°C–25°C) and human body temperature (37°C), and potentially back to the environment, during nosocomial infections inherently requires *P. aeruginosa* to adapt to different temperatures (Fig. 1).

## HOW CAN BACTERIA SENSE TEMPERATURE?

Like many bacteria, *P. aeruginosa* has evolved systems to sense virtually every aspect of its environment and accordingly adjust cellular processes to maintain homeostasis and survive. Genes related to the import and/or breakdown of certain sugars, metabolites, and important metals are often regulated by proteins that sense the presence or absence of that very molecule. For example, *P. aeruginosa* requires iron as a cofactor for many enzymes essential for survival, such as the cytochrome complexes (27). When iron (Fe^2+^) is present in the cytoplasm, it binds the transcriptional repressor Fur, and together Fur-Fe^2+^ repress the expression of genes whose products aid in the adaptation to environments with low, or no, iron, such as the human body (28–30). When Fe^2+^ is absent, the repressor becomes inactive, and genes are transcribed whose products help scavenge, sequester, and return iron to the cell.

**Fig 2:**
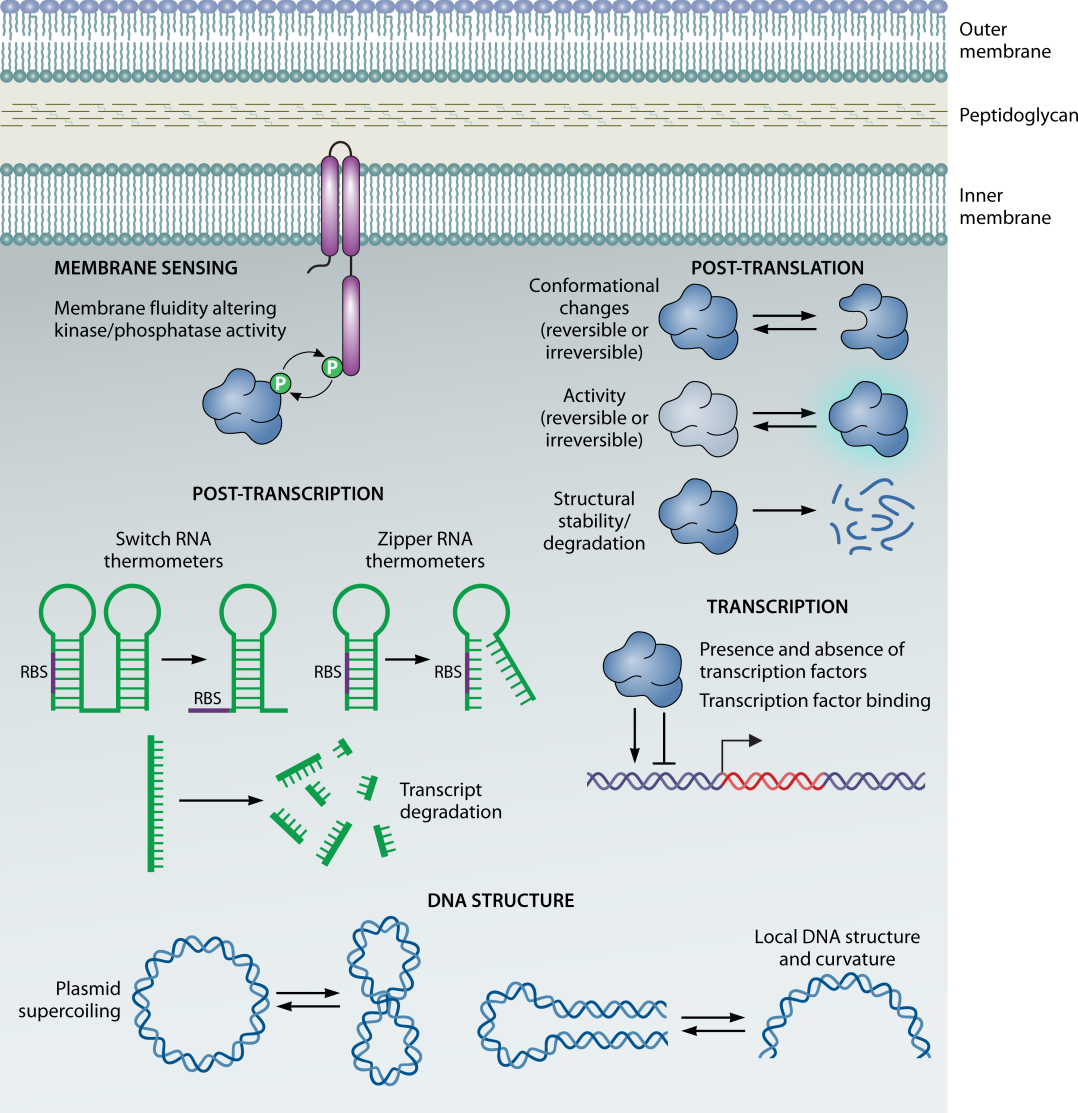
Temperature can affect many cellular processes in bacteria. A diagram of some bacterial cell processes that are known to be thermoregulated is shown. DNA structure: temperature can alter both plasmid supercoiling and the local structure of promoters to change the accessibility of DNA to transcriptional activators and repressors, as well as RNA polymerase, to ultimately affect transcription. Transcription: temperature can also affect transcription via controlling the presence (or absence) of transcription factors as well as their binding. Post-transcription: temperature can affect mRNA to ultimately thermoregulate protein levels through several mechanisms. Two types of RNA thermometers, switch and zipper thermometers, can possess temperature-sensitive secondary structures that modulate the accessibility of the ribosome binding site (RBS) to the ribosome. Transcripts can also be preferentially degraded in a temperature-dependent manner. Post-translation: temperature can change the structural conformation, activity, and stability of a protein. Changes to structure and activity may be reversible (can transition from “off” back to “on” state upon permissive temperature change) or irreversible (locked in “off” state by non-permissive temperature). Membrane sensing: increasing temperature increases membrane fluidity, while decreasing temperature decreases it. These changes can affect the structure of membrane kinases/phosphatases in two-component regulatory systems to control the corresponding response regulator(s).

In contrast, there has been no molecule identified that senses changes in temperature. Since temperature can directly affect virtually every aspect of bacterial physiology through biophysical changes (31), the study of adaptation to temperature can be broad and encompass many different types of thermoregulation (Fig. 2). For example, cells can sense alterations in temperature through changes in membrane fluidity. In general, bacterial (and archaeal) membranes become more fluid as temperature increases and less fluid as temperature decreases (32–34). Decreasing fluidity caused by cold conditions has been shown to alter the structure of membrane kinases to a more active form, leading to the phosphorylation of response regulators that upregulate genes that increase the amount of unsaturated fatty acids in membrane lipids (35–38). Transcription can be thermoregulated, which can be due to myriad mechanisms. Both transcriptional activators and repressors could be present or absent due to their own transcriptional thermoregulation or thermal (in)stability, thereby altering downstream gene expression in a temperature-dependent manner; regulators may also only be functional due to conformational changes occurring at certain temperatures, thus resulting in temperature-dependent regulation of their target genes (39, 40). The local structure of DNA, as well as the supercoiling of plasmid DNA, can also be affected by temperature changes and modulate the accessibility of promoters to transcriptional regulators and RNA polymerase (41, 42). The secondary structure of RNA can be sensitive to temperature and adopt different forms that either favor or hamper translation; RNA transcripts also can be preferentially degraded in a temperature-dependent manner, which ultimately affects translation (43). Finally, the structure, stability, or activity of a protein can all depend on temperature, which can result in the thermoregulation of the protein’s downstream effect—transcriptional regulators can thermoregulate gene expression, enzymes can produce temperature-dependent levels of products or post-translational modifications on other proteins, and so on (40, 44, 45). It is very likely that more mechanisms of thermoregulation exist than have currently been identified.

Advances in transcriptomics and proteomics have contributed significantly to the study of thermoregulation by facilitating non-biased screens of *P. aeruginosa* grown at different temperatures (46–50). A major benefit of omics techniques for studying thermoregulation is that the levels of RNA or protein in cells can be compared at two or more temperatures without selective pressure (e.g., transposon screening based approaches) to identify essential and non-essential thermoregulated genes and proteins. While transcriptomic and proteomic analyses have identified many thermoregulated genes and proteins, in many cases these studies have generated more questions than answers regarding the actual mechanisms of thermoregulation. The remainder of this review will summarize our current understanding of thermoregulation in *P. aeruginosa*, focusing on the different levels of thermoregulation (i.e., transcriptional, post-transcriptional, or post-translational), and highlight the gaps in the field’s mechanistic understanding of thermoregulation.

## OMICS STUDIES OF THERMOREGULATION

### Transcriptomics at room temperature versus 37°C

Three transcriptomic studies have characterized global gene expression at two temperatures relevant to *P. aeruginosa* as an opportunistic pathogen that survives in anthropogenic environments. The first one determined the transcriptome of the laboratory strain PAO1 grown to stationary phase at 25°C compared to 37°C by microarray, with attention to the genes upregulated at 25°C; at the time, many of the thermoregulated genes were of unknown function, but many genes related to fundamental cell functions, like metabolism and protein secretion, were upregulated at 25°C (46). To further explore secretion due to the importance to *P. aeruginosa* virulence, the authors also analyzed secreted proteins to correlate the transcriptome with the secretome. They found that levels of the secreted exoprotease PrpL (called PIV for protease IV in the remainder of this review) and the type VI secretion system effector Hcp were higher at 25°C in accordance with thermoregulated gene expression; interestingly, levels of the secreted protease AprA were also higher at 25°C despite gene expression not being affected by temperature (46). This early study provided insights into how *P. aeruginosa* may adapt to suboptimal temperatures in environments such as hospitals. The second transcriptome was determined by RNA-seq of another laboratory strain PA14 grown at 28°C compared to 37°C (47), and another was determined by microarray of PAO1 grown at 22°C compared to 37°C (48). The RNA-seq study of PA14 (47) found significant enrichment of type III secretion system genes and phenazine biosynthesis genes at 37°C, while protein synthesis genes were upregulated at 28°C. The single-nucleotide resolution of the PA14 RNA-seq study also allowed for the definition of transcription start sites and 5’ untranslated regions (UTRs), new LasR binding sites, and non-coding RNAs. The microarray of PAO1 (48) also found that protein synthesis genes were upregulated at 22°C, while most secreted factors and virulence factors were upregulated at 37°C with the exception of *piv,* as had been previously noted (46). Both these transcriptomic studies (47, 48) examined cells at stationary phase and identified hundreds of thermoregulated genes, with differences in the identity of the genes possibly due to different strains being used. Although both studies found that most virulence factors and many quorum sensing regulated genes are upregulated at 37°C while protein synthesis genes are upregulated at 25°C, the central findings and foci of the PA14 and PAO1 transcriptome studies differed largely. For example, the type III secretion system (T3SS) and phenazine biosynthesis genes were upregulated at 37°C in PA14 (47), while only a few T3SS effectors and one phenazine biosynthesis gene, *phzS*, were noted as upregulated at 37°C in PAO1 (48). In the PAO1 microarray study (48), the arginine deiminase and succinyltransferase, encoded by the *arcDABC* and a*ruCFGDBE* operons, were upregulated at 22°C but not thermoregulated in the PA14 RNA-seq study (47). The transcriptome of the rhizosphere strain *P. aeruginosa* M18 had also been determined at 28°C compared to 37°C by microarray (51). Similar to PA14, M18 genes related to mammalian virulence, such as T3SS and substrates, secreted proteases, and phenazine biosynthesis, were more highly expressed at 37°C than 28°C. Genes likely related to survival in the unique rhizosphere inhabited by M18, such as copper resistance genes *pcoA*/*pcoB* and aromatic compound metabolism genes, were upregulated at 28°C, a temperature more similar to that of the rhizosphere (51).

### Transcriptomics at 37°C versus heat shock

In addition to the change in temperature associated with transitioning from the environment to the human host, *P. aeruginosa* can also experience a change from average human body temperature, 37°C, to elevated temperatures mimicking fevers. One such transcriptomic study examined PAO1 grown at 37°C versus 46°C, a temperature more relevant to experimental heat shock (HS) conditions than physiological human fevers (49). Cultures grown to exponential phase at 37°C compared to 46°C (or 37°C as a control) for 30 min prior to RNA extraction and sequencing to characterize the global HS response. The expression of 133 genes was differentially regulated by HS, with well-known bacterial HS genes *clpB*, *dnaK*, *dnaJ*, *groEL*, *grpE*, and *asrA* highly upregulated at 46°C. An interesting finding was the HS upregulation of *rsmA*, a complex post-transcriptional regulator controlling many phenotypes, including the transition from acute to chronic infections (52, 53). The authors also noted a number of genes were downregulated at 46°C, including genes related to the type IV pili and type VI secretion system, which is not as often examined but represents an important aspect of how *P. aeruginosa* adapts to HS (49).

### Transcriptomics at 37°C versus febrile temperatures

*P. aeruginosa* and *Staphylococcus aureus* are each major causes of morbidity and mortality in CF patients, and co-infections with both leads to significantly worse patient outcomes (13, 14, 54). Another transcriptomic study examined how PAO1 and *S. aureus* USA300 responded to growing at 39°C, a physiologically relevant fever temperature, in coculture versus monoculture (50). When in monoculture, growth at 39°C compared to 37°C induced relatively few transcriptional changes in PAO1 compared to growth at 37°C; when in coculture with USA300, growth at 39°C compared to 37°C induced changes in the expression of PAO1 metabolic pathways, motility genes, and the type III secretion system. This work showed how multiple environmental factors, such as temperature and community composition, can interact to alter bacterial physiology.

Transcriptomic and proteomic studies have identified many thermoregulated genes and proteins for future mechanistic studies, and in many cases these have generated more questions than answers regarding mechanisms of thermoregulation. Transcriptomics can identify genes whose transcript levels are thermoregulated but do not distinguish between transcriptional thermoregulation or transcript thermal (in)stability or both as mechanisms for transcript level thermoregulation. Similarly, proteomics can identify proteins whose levels are thermoregulated, but further experiments are required to determine if this is because transcript levels are actually thermoregulated, a transcript is preferentially translated depending on temperature, and/or the protein’s structure is thermolabile. Coupling these techniques on the same samples could be a powerful tool to identify proteins whose abundance is thermoregulated, but gene expression is not, which would hint at a post-transcriptional or possibly post-translational mechanism of thermoregulation. Specific mechanistic studies, some of which are described in subsequent sections, are important for further characterizing the findings of omics studies.

## TRANSCRIPTIONAL THERMOREGULATION

Gene expression can be thermoregulated due to differences in promoter activity, which can result from temperature-sensitive transcriptional regulators and/or sigma factors. Transcriptional thermoregulation is often a mechanistic basis underlying differences in protein levels, as increased transcription at one temperature results in more translation of that gene product and is a common mechanism for bacteria to rapidly adapt to a stressful environment, such as HS. Recently, there have also been more studies of genes whose transcriptional thermoregulation is individual and not part of a centralized response such as the HS response, as well as genes upregulated at low versus high temperatures, which have been historically less well studied.

### Heat shock response regulation

An early study on HS in *P. aeruginosa,* informed by concurrent research on the *Escherichia coli* HS response, identified 17 proteins induced by shifting *P. aeruginosa* grown at 30°C to early exponential phase to 45°C for up to 10 min (55). Antibodies to known *E. coli* HS proteins were used to identify homologous proteins upregulated by HS in *P. aeruginosa*, such as DnaK and GroEL. Antibodies specific to *P. aeruginosa* RNA polymerase and sigma factors were also used to identify cross-reactive HS sigma factors, such as the principal sigma factor RpoD (σ^70^) and the then uncharacterized HS sigma factor RpoH (σ^32^). Subsequently, the *rpoH* gene was identified, sequenced, and found to complement *E. coli rpoH* mutants (56, 57). Naczynski et al. additionally identified transcription start sites under control versus HS conditions and corresponding promoter consensus sequences for RpoD (σ^70^) and RpoE (now known as AlgU or AlgT, also σ^E^ or σ^24^), which, at the time, were known to be required for HS induction of *rpoH* expression from its third promoter, *rpoH* P3 (58, 59). Expression of *rpoD* had been found to increase upon temperature shift to 42°C (60). Further characterization of its multiple promoters revealed this is due to RpoD directing its own transcription at both 30°C and 42°C from its constitutive promoter and at 42°C from its upstream HS promoter (61). RpoH also activates *rpoD* at 30°C and 42°C from only the HS promoter (61). Through these regulation studies, it was understood that increased levels of at least RpoH and RpoD proteins observed in early studies (55) were due to transcriptional thermoregulation and subsequently increased gene expression under HS conditions.

**Fig 3:**
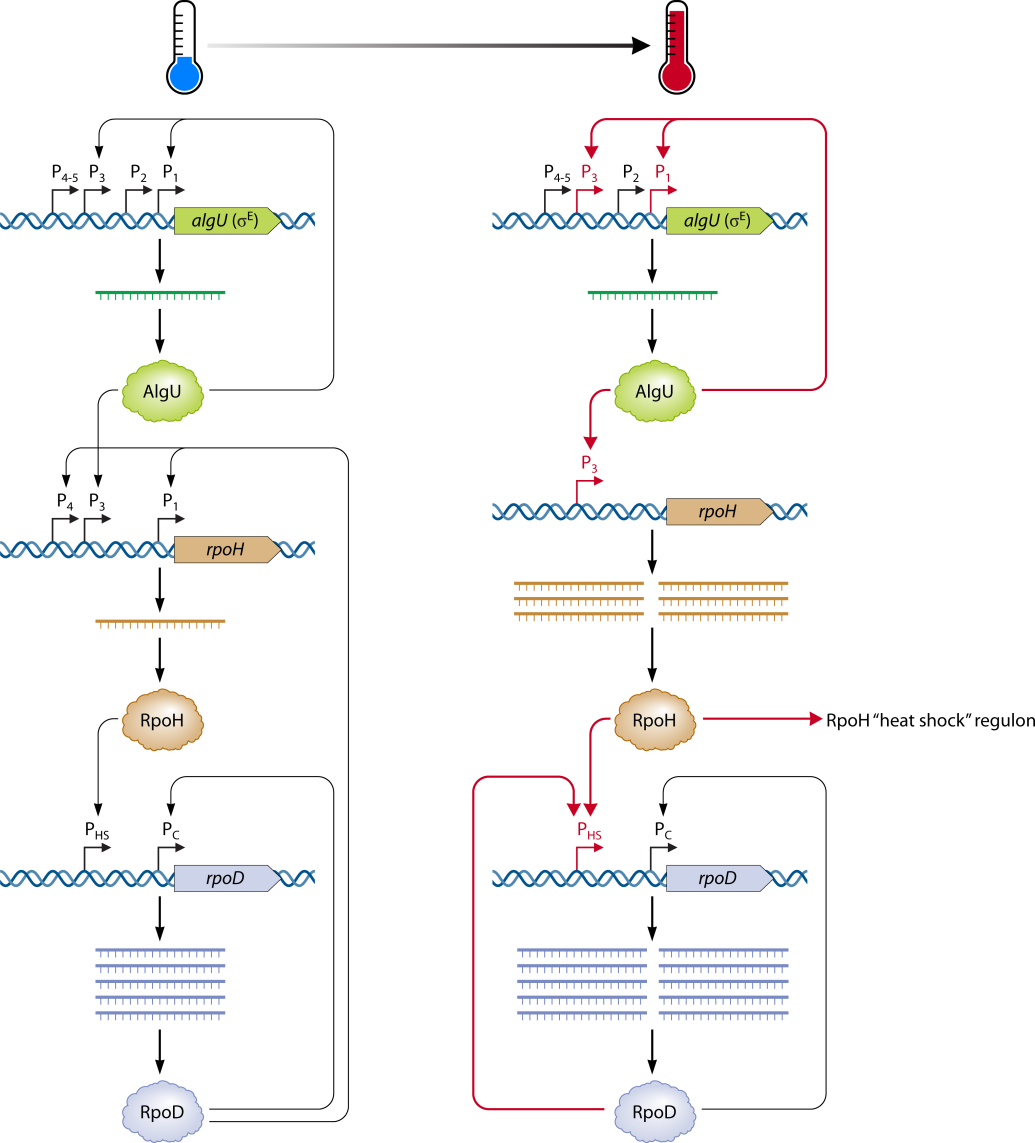
Induction of the HS response in *P. aeruginosa* depends on transcriptional thermoregulation. A diagram of the cascade of transcriptional thermoregulation underlying the HS response is shown. Under non-HS conditions (left), *algU* is constitutively transcribed at low levels. AlgU (also called AlgT or RpoE/σ^24^) upregulates itself from its first and third promoters (P_1_ and P_3_) and upregulates *rpoH* from its third promoter. RpoH directs transcription of *rpoD* from its HS promoter (P_HS_) even under non-HS conditions. In turn, RpoD (σ^70^) also transcribes itself from its constitutive promoter (P_C_) and *rpoH* from its first and fourth (P_4_) promoters. Under HS conditions (right), AlgU upregulates itself from its P_1_ and P_3_ and upregulates *rpoH* from its P_3_, the only *rpoH* promoter active under HS. RpoH activates its regulon of HS response factors and also upregulates *rpoD* from P_HS_. RpoD also directs its own transcription from P_HS_ while also continuing to do so from P_C_. Red arrows and promoters indicate those upregulated under HS.

Research on the regulation of mucoid conversion, which occurs when *P. aeruginosa* overproduces the exopolysaccharide alginate, has been of high interest due to the contribution of mucoidy to chronic infections and poor outcomes in CF patients. It was noted that AlgU (also called AlgT), the sigma factor that directs expression of the alginate biosynthetic genes, was highly similar and interchangeable with *E. coli* σ^E^ and sometimes referred to as the σ^E^ (or RpoE) of *P. aeruginosa* (62). This led to the discovery that *algU* expression increased under HS at 50°C from its first and third promoters, resulting in increased AlgU protein levels and increased expression of the AlgU-regulated *algR* gene in a nonmucoid PAO1 strain (63). The first and third *algU* promoters also strictly required AlgU itself for their activity. AlgU was subsequently found to direct transcription of *rpoH* from its HS-inducible *rpoH* P3 (64), which made a connection to previous research that the consensus sequences of *rpoH* P3 matched those of *E. coli* σ^E^ promoters (57) and solidified the notion from early HS studies that the alginate sigma factor AlgU is the same as the sigma factor referred to as RpoE (σ^E^) or σ^24^. Work on the AlgU regulon improved the understanding that HS induction occurs at the level of transcriptional thermoregulation (Fig. 3), wherein AlgU (σ^E^ of *P. aeruginosa*) is constitutively transcribed at low levels by RpoD; at HS temperatures (~50°C), AlgU increases transcription of itself and *rpoH*, each from a HS-specific promoter distinct from the constitutive promoter directed by RpoD (σ^70^). RpoH then directs transcription of a HS regulon and RpoD from its HS promoter. More recently, the primary *P. aeruginosa* RpoH regulon was determined by combining RNA-seq and chromatin immunoprecipitation coupled with next-generation sequencing (ChIP-seq); this analysis identified many proteases and protein chaperones recognized to be essential for cell viability at high temperatures (65).

While transcriptional thermoregulation of HS is perhaps the most studied mechanism of thermoregulation in *P. aeruginosa*, it also has the most open questions remaining with regard to how temperature is transduced to regulate gene expression. For example, it is now well understood that AlgU is upregulated from specific promoters under HS and proceeds to upregulate itself and RpoH, which in turn upregulates a suite of HS response genes, including DnaK and GroEL. However, how temperature induces *algU* transcription from HS promoters to begin the HS cascade, and how AlgU regulates itself from these same promoters as part of its thermoregulation, has not been determined. One possibility is the presence of an additional transcription factor activated by HS that promotes *algU* expression from HS promoters. Another possibility is a DNA thermometer, which has been relatively understudied in bacteria compared to other forms of thermoregulation, especially in *P. aeruginosa*. Akin to RNA thermometers (see “Post-transcriptional thermoregulation,” below), DNA thermometers are regions of often curved DNA whose structures are inherently sensitive to changes in temperature (66). One of the most well-studied DNA thermometers in pathogenic bacteria is the promoter of the master virulence factor regulator *virF* of *Shigella flexneri*, which adopts a curved structure below 32°C that allows repressors to bind; above 32°C, the DNA straightens out to hamper repressor binding and encourage *virF* transcription (41, 67, 68). A DNA thermometer within the promoter of *algU*, for example, could theoretically change the accessibility of transcription factor binding sites to ultimately modulate expression at different temperatures. DNA thermometers may play a so-far unappreciated role in transcriptional thermoregulation by modulating the accessibility of DNA to transcriptional regulators and RNA polymerase.

### Specific genes that show transcriptional thermoregulation

Benefitting from advances in RNA-seq, more unbiased studies on how temperature affects the global *P. aeruginosa* transcriptome (47, 48, 69) have helped identify genes whose expression is thermoregulated, especially at temperatures colder than 37°C. One such gene, *piv*, was consistently upregulated at the tested room temperature, 22°C or 28°C, compared to 37°C (47, 48). As PIV (protease IV, also called PrpL) is a secreted protease that degrades lactoferrin and transferrin (70) and contributes to virulence in corneal, lung, and burn wound models of infection (71–75), its upregulation at room temperature seemed unique. In brief, we found *piv* was thermoregulated at the level of transcription and that the quorum sensing regulator LasR upregulated *piv* much more at 25°C than at 37°C (76). Another virulence factor upregulated at room temperatures is the methyltransferase EftM (see “Post-translational thermoregulation,” below); *eftM* is more highly expressed at 25°C than 37°C due to increased promoter activity at 25°C (77).

One of the early RNA-seq studies of *P. aeruginosa* grown at different temperatures compared the transcriptome at 37°C to 46°C, a HS condition, and identified *cmaX* as a HS-induced gene (49). *cmaX* is the first gene in the *cmaX-crfX-cmpX* operon, which is predicted to contain transmembrane proteins. Although the function of *cmaX-crfX-cmpX* in helping the cell adapt to different thermal stressors is unclear, a subsequent study of the complex transcriptional thermoregulation of the operon provided preliminary insight into how membrane fluidity may sense and respond to temperature changes, particularly cold shock (78). *cmaX* was most upregulated by HS at 60°C due to increased transcription requiring AlgU (see previous discussion of heat shock response regulation), while *crfX* and *cmpX* were more responsive to cold shock (CS) at 4°C and transcriptionally upregulated by SigX from a distinct promoter. SigX is an alternate sigma factor that responds to cell envelope stress and can increase survival of cells under CS but not HS, possibly by regulating fatty acid biosynthesis genes to increase the incorporation of shorter fatty acids into the membrane to increase membrane fluidity under cooler conditions (79).

Another thermoregulated gene predicted to be involved in membrane adaptation to temperature change is *htrB1* (PA0011), a 2-OH-lauroyltransferase involved in lipid A biosynthesis that presumably controls acyl chain fluidity of lipid A molecules of lipopolysaccharide (LPS) anchored in the outer membrane (80). The authors found that *htrB1* was upregulated at 21°C compared to 37°C and accordingly was more important for resistance to cell wall inhibiting carbapenems at 21°C than 37°C, although a mechanism for transcriptional thermoregulation was not further characterized (80).

Although it is not well understood how changes to membrane fluidity caused by different types of stress are detected by the cell, membrane fluidity stress is known to alter gene expression and cell physiology. A mutant with constitutively reduced membrane fluidity showed increased expression of the stringent response regulator RelA, which resulted in increased levels of the alarmone (p)ppGpp and caused premature activation of quorum sensing (81). Vancomycin can decrease membrane fluidity and induce *sigX* expression in *P. aeruginosa*, as does CS (33, 34). Together, these findings hint that an unknown transmembrane protein could be structurally and/or functionally altered by decreases in membrane fluidity in a manner that results in the activation of regulators like SigX, which controls gene expression to increase membrane fluidity and adaptation to stressful environments.

The production, structure, and organization of biofilms in multiple strains have also been shown to be affected by temperature at the level of transcriptional thermoregulation (82, 83). One study examined biofilm formation at 20°C, 25°C, 30°C, and 37°C in PAO1 and PA14 and found that biofilm formation was highest at 20°C, then 37°C, then 30°C, and lowest at 25°C (82). Biofilms formed at 20°C were also thickest and formed a unique mushroom-like structure absent at higher temperatures, even as the total biomass and thickness increased at 30°C and 37°C compared to the lowest values observed at 25°C. The authors found levels of intracellular cyclic diguanylate (c-di-GMP), a secondary messenger molecule that regulates biofilm formation through multiple mechanisms (84), peaked at 20°C, as did the transcription of the biosynthetic genes for the important exopolysaccharides (EPS) alginate, Pel, and Psl in PAO1 and PA14 (82). This likely explains the peak mass of biofilms found at 20°C. The exact thermoregulation pattern of biofilm formation, c-di-GMP levels, and EPS gene expression varied between PAO1 and PA14 at 25°C, 30°C, and 37°C, suggesting strain-specific differences in how temperature is sensed to regulate biofilm formation. Interestingly, a clinical isolate of *P. aeruginosa* has been found with increased biofilm formation at 37°C due to increased c-di-GMP synthesis (85), underscoring the variation in biofilm formation and regulation between *P. aeruginosa* strains (see “Post-translational thermoregulation,” below). Another study of only PA14 found that expression of the filamentous prophage Pf1 was higher at 37°C than at 23°C, and loss of the major Pf1 coat protein gene *coaB* resulted in decreased biofilm mass only at 37°C with no effect at 23°C (83). As this filamentous prophage (called Pf4 in PAO1) has also been linked to disease severity phenotypes via modulating innate immunity and promoting biofilm dispersal during infections (86–89), its transcriptional upregulation at 37°C suggests this thermoregulation is an adaptive mechanism for mammalian infection.

The mechanistic underpinning of transcriptional thermoregulation is difficult to study; unlike regulators that respond to specific environmental or nutrification cues, such as the alternate sigma factor PvdS responding to low iron or the quorum sensing regulator LasR activated at high cell densities, there is no one specific consensus sequence that has been recognized for thermoregulated transcription factor binding, which hampers bioinformatic study. Newer next-generation sequencing techniques like ChIP-seq or ChIP-qPCR could allow for the identification of promoters with temperature-sensitive transcription factor binding and improve our understanding of how *algU* is thermoregulated, how LasR thermoregulates *piv*, and others. More studies of how *P. aeruginosa* systematically responds to CS would also be beneficial, as virtually all studies have focused on HS.

## POST-TRANSCRIPTIONAL THERMOREGULATION

Another mechanism for bacteria to rapidly respond to changes in temperature is an RNA thermometer (RNAT), a secondary structure that controls translation of a transcript. Generally, RNATs are found in the 5′ UTR of an mRNA and function by forming a secondary structure that occludes the ribosome binding site (RBS) at the non-permissive temperature(s) but adopts an RBS-accessible structure at the permissive temperature(s) (90). There is little to no sequence conservation of RNATs, which presents a challenge for bioinformatic identification, although great efforts have been made toward computational algorithms utilizing structural predictions to identify RNATs (91–93).

Most research has been conducted on RNATs that “open,” sometimes instantaneously, at warm temperatures to promote the translation of virulence factors relevant to mammalian infection (94, 95). This has led to the characterization of three categories of “zipper-like” RNATs based on the features of the secondary structure occluding the RBS.

- The ROSE (repression of heat shock gene expression) family, characterized by a long (60–120 bps or longer) 5′ UTR with two to four often complex loops and a crucial hairpin near the 3′ end that occludes the RBS. Within that hairpin, and on the side opposite to the RBS, is an unpaired G that is important for the unzipping of the hairpin in response to increasing temperature. RNATs with some but not all the characteristics of the first ROSE-element identified in *Bradyrhizobium japonicum* are referred to as ROSE-like (96, 97).
- The fourU family, characterized by a stretch of four uridines that pair with a portion of the RBS, often but not always the sequence AGGA (98).
- The uncategorized family, which resembles neither ROSE nor fourU RNATs and may rely more on canonical base pairing or small hairpins or loops (99–101).

Zipper-like RNATs gradually “melt,” or move toward open conformation, as temperature increases and destabilizes the base pairing comprising the secondary structure. In contrast with zipper-like RNATs, switch RNATs adopt two distinct secondary structures, one “open” and one “closed” to translation, depending on temperature. These have been found in the 5′ UTR of CS genes and the best documented bacterial example is the 5′ UTR of the transcript encoding the RNA chaperone cold shock protein A (CspA) of *E. coli*, which adopts a secondary structure exposing the RBS for translation at cold temperatures and a distinct structure occluding it at 37°C (102). The bacterial RNAT field has been extensively reviewed (43, 90, 94, 95, 103, 104) and for the sake of this minireview we will focus more on the types of RNATs that have been documented in *P. aeruginosa* (Table 1), with a discussion of what has not been identified or has potential for further characterization in this bacterium. The predicted secondary structures and experimental validations for each of the following RNATs can be found in the referenced manuscripts (Table 1), and we encourage interested readers to refer to them.

**TABLE 1:** Characterized RNATs in *P. aeruginosa*

Gene	RNAT type	Key features	Reference(s) for experimental studies and structural predictions
*rhlA*	ROSE	Anti-RBS sequence occludes the RBS	105
*lasI*	ROSE-like	Anti-RBS sequence partially occludes the RBS	105
*ibpA*	ROSE-like	Anti-RBS sequence occludes the RBS	106, 107
*ptxS*	Unclassified	Stem-loop occludes translation initiation regions; no canonical sequence elements	108
PA5194	fourU-like	threeU sequence partially occludes the RBS	108

### ROSE and ROSE-like RNATs

Transcriptomics of *P. aeruginosa* at room versus human body temperature have revealed that many quorum sensing regulated genes are upregulated at 37°C (47, 48). Quorum sensing upregulates the expression of genes encoding many virulence factors and secreted public goods and has long been of great interest to the *P. aeruginosa* field (109). In laboratory strains of *P. aeruginosa*, multiple quorum sensing systems are arranged hierarchically and have overlapping but also distinct regulons (110). Relevant to the studies discussed here, the master LasRI system regulates itself and a negative regulator, *rsaL,* via the divergent *lasI*/*rsaL* promoter; the elastase gene *lasB*; and the RhlRI quorum sensing system (111, 112). Regulation of RhlRI is slightly more complex, as RhlR can be transcribed from four different promoters directly upstream of its coding region or as part of a polycistronic mRNA originating from the upstream *rhlAB* operon promoter that reads through due to an apparent lack of terminator following *rhlAB* (113). Thus, the RhlRI system can regulate itself via the polycistronic transcript originating from the *rhlAB* promoter.

An RNAT with a ROSE element was identified in the 5′ UTR of *rhlA* and found to thermoregulate RhlR protein levels when *rhlR* was expressed in the polycistronic *rhlAB-R* transcript (105). The *rhlA* RNAT was over 100 bp in length and structural predictions revealed multiple large loops, including a hairpin containing the RBS near the 3′ end of the UTR. Interestingly, the anti-RBS diverged from the U(U/C)GCU sequence typical of ROSE thermometers but did retain the important unpaired G opposite the RBS. The authors found that improved translation of the *rhlAB-R* transcript at 37°C increased transcriptional readthrough, which ultimately increased RhlR protein levels at 37°C, although the mechanism by which this presumed polar effect acts remains to be elucidated. The unique effects of this RNAT on RhlR levels contribute to increased levels of RhlR-regulated virulence factors at 37°C, including rhamnolipids themselves as well as pyocyanin (105).

An RNAT with some features of a ROSE element was found in the 5′ UTR of *lasI*; half of the RBS was occluded by a hairpin and the other half was exposed in a single-stranded loop, and the hairpin lacked the characteristic unpaired G across of the RBS (105). Based on these characteristics, the RNAT was deemed ROSE-like, although it perhaps more resembles an uncategorized RNAT, as the unpaired G characteristic of a ROSE element is critical for its highly temperature-sensitive unzipping mechanism (97). Although this structure did contribute to *lasI* thermoregulation, the effect on LasRI-regulated genes aside from *rhlRI* was marginal. Another ROSE-like RNAT was bioinformatically predicted in the 5′ UTR of the HS protein *ibpA* (106) and the mechanism by which temperature alters secondary structure to modulate ribosome accessibility was well-characterized (107). The short 61 bp RNAT has two loops, the second of which was shown to contain a hairpin occluding the RBS with the ROSE typical anti-RBS sequence U(U/C)GCU and the central unpaired G. The RNAT exerted translational control by blocking ribosome access at 25°C but allowing it at 42°C, which was demonstrated by toeprinting assays. Structural probing using RNases also supported the RBS-accessibility model for RNAT melting, where increased temperature physically opened the hairpin to expose the RBS. Only the structure of the second hairpin containing the RBS was affected by temperature and this was sufficient to act as the RNAT controlling translational thermoregulation. Due to the small size, the lack of importance of 5′ loops, and general structural simplicity, this RNAT was deemed ROSE-like.

### Uncategorized RNATs

As mentioned, systematic identification of RNATs is difficult due to the lack of conserved sequences. A clever forward genetic selection identified four gene candidates for post-transcriptional thermoregulation whose “on” state was at 37°C (108). The 5′ UTRs of *ptxS* and PA5194, a putative membrane protein, were validated as controlling post-transcriptional thermoregulation when *P. aeruginosa* transitioned from 28°C to 42°C. The predicted secondary structures did not resemble ROSE or fourU elements; the *ptxS* RNAT contains two stem-loops, while the PA5194 is a more complex structure with four loops. RNase degradation and mutational analysis suggest the second loop of the *ptxS* RNAT, containing translational initiation regions, opens at 42°C. The PA5194 RNAT has both the RBS and the AUG start codon within the second hairpin; half of the RBS is predicted to engage in base pairing with a UUU anti-RBS reminiscent of fourU RNATs. Mutations to this threeU anti-RBS did not completely ablate thermal response, but rather tuned overall translation levels, suggesting that this RNAT may function by modulating accessibility of both the RBS and the start codon in response to temperature. To the best of our knowledge, a canonical fourU RNAT has not been identified in *P. aeruginosa*, and although the PA5194 RNAT does not match all the fourU characteristics, we find it notable that it has fourU-like elements.

It can be difficult to identify, and thus study, RNATs (114). Advances in using structural modeling to predict RNA structure in response to temperature have been made and aided in the identification of new RNATs (91–93) but are still limited in their ability to identify novel types of RNATs that do not fit ROSE or fourU elements and/or RNATs that rely heavily on non-Watson-Crick base pairing. Techniques like selective 2′-hydroxyl acylation analyzed by primer extension and mutational profiling (SHAPE-MaP) (115), which couple chemical-based RNA structural analysis with next-generation sequencing, could theoretically be applied to unbiasedly determine all the RNAs with temperature-sensitive structures in a specific bacterium of interest. A benefit of techniques like SHAPE-MaP is that experiments can be conducted on living cells growing at different conditions, such as temperatures, thus making it possible to investigate the effect of temperature on the structure of RNAs *in vivo* (115).

As with other forms of thermoregulation, there are generally more studies of RNATs that “open” at warmer temperatures across bacterial species, and especially in *P. aeruginosa*. For example, the “Tet-Trap” system was applied to search for 5’ UTRs containing RNATs in the ‘on’ state at 37°C (108), although it could also presumably be used to search for 5′ UTRs that are in an “on” state at lower temperatures. The term RNAT is sometimes used to refer only to thermometers whose permissive state is achieved as temperature increases, despite several known thermometers whose permissive state is at cold temperatures or favored as temperature decreases to control translation (102, 116). We encourage broadening the term RNAT to an RNA with temperature-sensitive secondary structure(s), with further subcategories of RNATs characterized by the mechanism by which the thermometer transduces temperature and the temperature of the permissive state. Because CS thermometers are accessible for translation as temperature decreases, their mechanisms of action are often different from HS thermometers (90), and more studies of them could identify novel types of thermometers as well as thermometers in genes not related to HS or virulence, of which much less is known.

## POST-TRANSLATIONAL THERMOREGULATION

The activity of enzymes is generally affected by temperature, but beyond that, the structure, stability, and post-translational modification (PTM) of a protein can all be affected by temperature, which ultimately thermoregulate protein activity. Like other mechanisms of thermoregulation, identification of post-translationally thermoregulated proteins often involves laborious screens for specific phenotypes, large proteomics studies, or serendipitous discovery.

### Thermosensitive protein structures

Many microbes, including respiratory pathogens, present phosphorylcholine (ChoP) on the cell surface to mimic the platelet-activating factor (PAF) recognized by the eukaryotic PAF receptor and to promote bacterial adhesion and colonization, particularly of epithelial cells (117). Discovery of the thermoregulated methyltransferase EftM began with the identification of a ChoP-antibody recognized epitope on elongation factor Tu (EF-Tu) that was displayed on the bacterial cell surface and present more at 22°C than 37°C (118). A screen of the PA14 transposon library for mutants lacking this PTM on EF-Tu at any temperature led to the identification of *eftM* (EF-Tu-modification enzyme), which was recapitulated in a PAO1 background (119). Mass spectrometry and biochemical assays revealed the ChoP-like epitope was actually the addition of three methyl groups to the lysine residue 5 of EF-Tu by EftM and that this trimethylation mimics the structure of both ChoP and PAF to interact with the PAF receptor on airway epithelial cells and facilitate attachment (119); interestingly, this trimethylation does not affect the canonical function of EF-Tu as a carrier of aminoacylated tRNAs to the ribosome complex during translation (77). EftM trimethylation activity occurred at 25°C but not 37°C *in vivo,* and incubation of purified EftM protein at 37°C significantly reduced enzymatic activity *in vitro*, as the structure of EftM is highly thermolabile and irreversible unfolding occurs at 30°C and higher (120). Immunological insights combined with biochemical characterization of EftM contributed to the hypothesis that trimethylation of EF-Tu at room temperature aids in the initial adaptation to the mammalian host from the environment by priming *P. aeruginosa* for epithelial attachment (119).

### Thermosensitive protein activity

Activity of the thermosensitive diguanylate cyclase TdcA, identified in the chronic CF isolate CF39S, was found to be thermoregulated (85). The authors found that CF39S produced biofilms much more as the temperature increased, with maximum biomass achieved at 34°C and above, due to TdcA producing increasing levels of c-di-GMP. TdcA is absent in laboratory strains of *P. aeruginosa* but is present in clinical strains within a Tn*7*-like transposon that resembles the horizontally acquired locus of heat resistance first identified in *E. coli*. TdcA was predicted to contain an N-terminal Per-Arnt-SIM (PAS) sensory domain connected to a well-characterized glycine–glycine–aspartate–glutamate–phenylalanine (GGDEF) c-di-GMP catalytic domain by an α-helical linker. Extensive characterization demonstrated that the PAS domain senses temperature and likely regulates catalytic activity of the GGDEF domain through PAS structural changes transduced via the α-helical linker. The thermoregulated c-di-GMP levels not only affect biofilm production but also the virulence of *P. aeruginosa* at ambient versus human body temperatures (85).

The thermosensitive methyltransferase EftM and the diguanylate cyclase TdcA are rather well-characterized in terms of how the thermal signal is actually transduced to regulate protein activity and the role that thermoregulation of these proteins may play in *P. aeruginosa* pathogenesis at different stages of infection. Interestingly, the modification created by EftM is conserved across a selection of genetically unrelated clinical strains studied (118), while TdcA was found in less than 1% of *P. aeruginosa* sequences available in the *Pseudomonas* Genome Database (85). The acquisition of TdcA may enable *P. aeruginosa* strains to form chronic infections by increasing biofilm production in response to the temperature of the human host. Overall, fewer mechanisms of post-translational thermoregulation have been identified than the other types presented in this review. More studies of proteins whose structures, stability, and/or activity are thermoregulated with thorough mechanistic characterization would greatly advance the field.

## *P. AERUGINOSA* IS A UNIQUE BACTERIUM FOR STUDYING THERMOREGULATION

Understanding how temperature regulates aspects of its physiology, particularly phenotypes relevant to pathogenesis such as quorum sensing, biofilm formation, and virulence factor regulation, is fundamental for understanding how *P. aeruginosa* successfully transitions from living in an ambient environment, such as a contaminated hospital surface, to living inside a human host. Although it is not an extremophile, its ability to survive across a broad range of temperatures is reflective of its adaptability, facilitated at least in part by regulatory responses. The virulence of many human pathogens, such as *S. flexneri* (67, 121), *Salmonella enterica* serovar Typhimurium (122), *Listeria monocytogenes* (123), and *Yersinia* spp., including *Y. pestis* (39, 124, 125), is strictly regulated by temperature by a master virulence regulator that is only active above 30°C. In contrast, our current understanding of thermoregulation in *P. aeruginosa* does not suggest that there is a centralized mechanism for transcriptional thermoregulation, such as a master transcriptional regulator responsible for activating genes required for virulence or other phenotypes. Rather, it seems that thermoregulated genes each have their own mechanism for thermoregulation. We believe this reflects the nature of *P. aeruginosa* as a generalist that can survive at a broad range of temperatures, rather than a mammalian or even human-specific pathogen. This makes *P. aeruginosa* an interesting bacterium in which to study thermoregulation, as there are potentially unique mechanisms of thermoregulation beyond a master regulator that underlie the temperature-dependent expression of hundreds of genes, as well as uncharacterized mechanisms for post-transcriptional and post-translational thermoregulation.
